# Investigation into the Dynamic Stability of Nanobeams by Using the Levinson Beam Model

**DOI:** 10.3390/ma16093404

**Published:** 2023-04-26

**Authors:** Youqin Huang, Richeng Huang, Yonghui Huang

**Affiliations:** Research Centre for Wind Engineering and Engineering Vibration, Guangzhou University, Guangzhou 510006, China; 2112016135@e.gzhu.edu.cn (R.H.); huangyh@gzhu.edu.cn (Y.H.)

**Keywords:** dynamic instability, nanobeams, Levinson beam, elastic medium, Bolotin’s method, parametric analysis

## Abstract

Dynamic stability is an important mechanical behavior of nanobeams, which has been studied extensively using the Euler–Bernoulli and Timoshenko beam theories, while the Levinson-beam-theory-based dynamic instability analysis of nanobeams has not been investigated yet. Shear deformation is not or is not suitably considered in the Euler–Bernoulli and Timoshenko theories, so it is very important to introduce the Levinson beam theory in the dynamic stability analysis of nanobeams, which correctly models the combined action of bending and shear in nanobeams with smaller length/height ratios. In this work, the equation of the transverse vibration of a Levinson beam embedded in an elastic foundation is firstly formulated based on the displacement field of Levinson beam theory, and the nonlocal theory is further applied to the Levinson nanobeam. Then, the governing equation of the dynamic stability of the Levinson nanobeam is derived using Bolotin’s method to achieve a generalized eigenvalue problem corresponding to the boundaries of regions of dynamic instability. The principal instability region (PIR) is the most important among all regions, so the boundary of the PIR is focused on in this work to investigate the dynamic stability of the Levinson nanobeam. When the width, length/height ratio, density, Young’s modulus, Poisson’s ratio, size scale parameter, and medium stiffness increase by about 1.5 times, the width of the PIR changes by about 19%, −57%, −20%, 65%, 0, −9%, and −11%, respectively. If a smaller critical excitation frequency and narrower width of the PIR correspond to the better performance of dynamic stability, the study shows that the dynamic stability of the Levinson nanobeam embedded in an elastic medium improves under a larger length and density and a smaller width, height, and Young’s modulus, since these factors are related to the natural frequency of the nanobeam which controls the width of the PIR. Additionally, the local model would overestimate the dynamic stability behavior of the Levinson nanobeam.

## 1. Introduction

Their excellent mechanical and electrical properties make nano-composite structures widely used in engineering fields [1,2,3,4]. The performance of dynamic stability is one of the crucial characteristics of nanobeams [5,6]. In the numerical analysis of the mechanical behavior of nanobeams, the method of atomistic simulations is time-consuming and the continuum theory is more efficient and popular. Since conventional local continuum elasticity does not include the quantum effects owing to the discrete nature at the nanoscale, the nonlocal continuum theory was proposed by Eringen [7,8] for the analysis of the continuum features of nanobeams.

Eringen’s theory has been widely applied in the dynamic stability analysis of nanobeams. Ghadiri and Hosseini [9] studied the nonlinear vibration and dynamic stability of Euler–Bernoulli nanobeams subject to thermo-magneto-mechanical loading. Sourani et al. [10] investigated the dynamic instability of a Euler–Bernoulli nanobeam under the action of axial dynamic excitation considering surface stress effects. It is known that the Euler–Bernoulli beam theory is only suitable for slender beams, while for beams with small length/height ratios whose shear deformation effects cannot be neglected, the Timoshenko beam theory is usually applied. Ansari and Gholami [11] carried out the dynamic instability analysis of nanobeams embedded in an elastic medium and thermal environment using the Timoshenko beam theory, and the effects of static load factor, size scale parameter, and medium spring constant were evaluated. Saffari et al. [12] explored the dynamic instability of functionally graded nanobeams under axial and thermal loading using the nonlocal Timoshenko model, and the influence of gradient index on the dynamic instability region was discussed. Hashemian et al. [13] paid attention to the dynamic instability of a Timoshenko nanobeam on an elastic foundation with the intermittent movement of nanoparticles. 

The Timoshenko theory considers the shear deformation effect of the beam by assuming a constant shear strain along the height of the beam through a shear correction factor, and the fluctuation of the shear correction factor with the length/height ratio would decrease the computational accuracy, so higher-order shear deform theories are desired by defining higher-order variations in shear strain over the height. Reddy [14] presented nonlocal forms of higher-order Reddy and Levison beam models, and they are now widely applied in the mechanical analysis of nanostructures [15,16]. 

The Levinson beam theory not only considers the combined effect of bending and shear, avoiding the errors caused by the Euler beam model for not involving the shear deformation, but also correctly considers the stress-free conditions of the upper and lower surfaces of the beam while maintaining the parabolic shear strain distribution. It does not require shear correction coefficients and decreases the inaccuracy owing to the fluctuations of shear correction coefficients. 

Iwase and Hirashima [17] applied the Levinson beam theory to treat the bending problems of thick regular beams with bimodulus materials. Li et al. [18] investigated the critical buckling loads of Levinson beams with functionally graded material, where a quadratic variation in transverse shear strain through the height was assumed. Li and Wan [19] gave the analytical results of the deflection, rotational angel, bending moment, and shear force of the Levinson beams with functionally graded material. Karttunen and von Hertzen [20] developed the exact Levinson beam finite element and a consistent variational formulation of the Levinson beam. Wand and Li [21] analyzed the natural frequency of the Levinson beam with functionally graded material and solved the two-point boundary value problem using a shooting method. Kryskoa et al. [22,23,24] used a size-dependent Levinson beam and the couple stress theory to investigate the regular and chaotic vibration of micro-beams and compared the results with those from the Euler–Bernoulli model and the Timoshenko model, discussing the effect of geometric and physical nonlinearity and the size dependency and the different performances of the variational process such as phase portraits, wavelet spectra, Fourier spectra, Poincare maps, and the largest Lyapunov exponents. Golbakhshi et al. [25] proposed a modified couple stress model based on the Levinson beam model to evaluate the bending and free vibration of functionally graded porous isotropic microbeams. 

However, the studies on the dynamic stability of nanobeams based on the nonlocal Reddy and Levinson beam theories are very limited. Huang et al. [26] derived the formulations of the dynamic instability of nanobeams according to the Reddy beam model and the Bolotin method, while the Levinson beam theory has not been applied in the dynamic stability analysis of nanobeams. The Lyapunov stability theory is effective for a homogeneous, nonlinear system, while the Bolotin method is popular for linear structures [27,28,29]. Consequently, the main contribution of this work is to provide a more accurate evaluation method for the dynamic stability of nanobeams based on the Bolotin method and the Levinson beam model which would avoid the errors caused by the Euler–Bernoulli and Timoshenko beam theories. In this paper, the formulations of Levinson’s nanobeam embedded in an elastic foundation are derived using Eringen’s nonlocal theory. Subsequently, the eigenvalue problem corresponding to the boundaries of dynamic instability regions are built according to the Bolotin method. The effects of various geometric and material parameters on the boundaries of dynamic stability are carefully explored via numerical computations. 

## 2. Formulation of Dynamic Stability of Levison Nanobeam

### 2.1. Transverse Vibration of Embedded Levinson Beam

Levinson proposed the shear deformation theory for rectangular beams by assuming that the axial displacement of any point on the cross section is a cubic function of the height of beam section and the first-order shear deformation theory [30]. 

**Figure 1:**
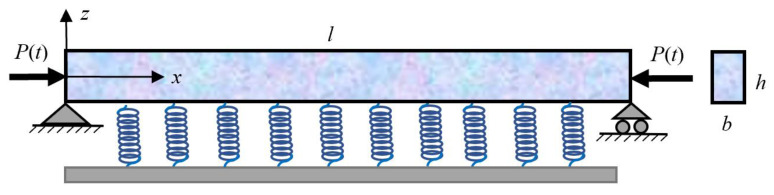
The mechanical model of Levinson’s nanobeam.

A nanobeam model considered as a Levinson beam is shown in Figure 1, which has the length *l*, and the rectangular cross section with height *h* and width *b*. The Levinson nanobeam has the material properties of mass density *ρ*, Young’s modulus *E,* and shear modulus *G*, where *G* = *E*/2(1 + *v*) and v is the Poisson’s ratio. The nanobeam is resting on an elastic foundation with the spring constant *q* and is subjected to axial harmonic excitation *P*(*t*). A coordinate system is taken on the nanobeam with the axis *x* along the nanobeam and the axis *z* perpendicular to the nanobeam. 

The expression of the displacement field is very important when deriving the vibration equation of the Levinson beam. The governing equation of the transverse vibration of the Levinson beam embedded in an elastic foundation can be derived by using the displacement field of the Levinson beam given by Reddy [14]

(1) $$u_{1}^{L}=u^{L}(x,t)+z\varphi^{L}(x,t)-c_{1}z^{3}\left(\varphi^{L}+\frac{\partial w^{L}(x,t)}{\partial x}\right)$$

(2) $$u_{2}^{L}=0$$

(3) $$u_{3}^{L}=w^{L}(x,t)$$

where ${\left(u_{1},u_{2},u_{3}\right)}^{L}$ denote the displacements along the coordinate axis *x*, *y*, and *z*, and the superscript *L* denotes the Levinson beam; $u^{L}$ and $w^{L}$ denote the displacements on the mid-plane (*z* = 0) along the axis *x* and *z*, and $\varphi^{L}$ is the shear deformation; $c_{1}$ is a constant, $c_{1}=4/\left(3h^{2}\right)$.

Furthermore, the longitudinal normal strain $\varepsilon_{xx}^{L}$ is the first derivative of the axial displacement $u_{1}^{L}$ along the *x* axis relative to *x*, and the transverse shear strain $\varepsilon_{xz}^{L}$ is the sum of the first derivative of the transverse displacement $u_{3}^{L}$ to *x* and the axial displacement to *z*, i.e.,

(4) $$\varepsilon_{xx}^{L}=\frac{\partial u_{1}^{L}}{\partial x}$$

(5) $$\varepsilon_{xz}^{L}=\frac{\partial u_{3}^{L}}{\partial x}+\frac{\partial u_{1}^{L}}{\partial z}$$

Thus, the corresponding strain field can be achieved as

(6) $$\varepsilon_{xx}^{L}=\frac{\partial u^{L}(x,t)}{\partial x}+z(1-c_{1}z^{2})\frac{\partial \varphi^{L}}{\partial x}-c_{1}z^{3}\frac{\partial^{2}w^{L}(x,t)}{\partial x^{2}}=\varepsilon_{xx}^{0}{}^{L}+zk^{L}+z^{3}\lambda^{L}$$

(7) $$2\varepsilon_{xz}^{L}=(1-c_{2}z^{2})\left(\frac{\partial w^{L}(x,t)}{\partial x}+\varphi^{L}\right)=\gamma^{L}+z^{2}\eta^{L}$$

where $\varepsilon_{xx}^{0}{}^{L}$ denotes the longitudinal normal strain on the mid-plane and $k^{L}=\frac{\partial \varphi^{L}}{\partial x}$, $\lambda^{L}=-c_{1}\left(\frac{\partial \varphi^{L}}{\partial x}+\frac{\partial^{2}w^{L}}{\partial x^{2}}\right)$, $\eta^{L}=-c_{2}\left(\varphi^{L}+\frac{\partial w^{L}}{\partial x}\right)$, $\gamma^{L}=\varphi^{L}+\frac{\partial w^{L}}{\partial x}$, $c_{2}=\frac{4}{h^{2}}$ For the Levinson beam resting on an elastic medium, its equation of transverse vibration can be written by referring to the standard formulations given by Reddy [14]:

(8) $$-m_{0}\frac{\partial^{2}w^{L}}{\partial t^{2}}+\frac{\partial Q^{L}}{\partial x}-P\frac{\partial^{2}w^{L}}{\partial x^{2}}+qw^{L}=0$$

(9) $$\frac{\partial M^{L}}{\partial x}-Q^{L}-m_{2}{}^{L}\frac{\partial^{2}\varphi^{L}}{\partial t^{2}}=0$$

where $M^{L}$ and $Q^{L}$ denote the bending moment and shear force on the cross-section, respectively; $qw^{L}$ is the force of elastic foundation; $m_{0}=\rho A$, $m_{2}{}^{L}=\frac{4}{5}\rho I$, and I is the sectional moment of inertia.

### 2.2. Vibration Equation of Nonlocal Levinson Beam

For the nonlocal nanobeam, the stress at a given point is affected by the strains of all points in the structure, that is [7,31]

(10) $$\sigma_{ij}(x)=\int \alpha (\left|x-x^{\prime }\right|,\tau )C_{ijkl}\varepsilon_{kl}(x^{\prime })dV(x^{\prime })$$

(11) $$\sigma_{ij,j}=0$$

(12) $$\varepsilon_{ij}=\frac{1}{2}(u_{i,j}+u_{j,i})$$

where $\sigma_{ij}$ and $\varepsilon_{ij}$ represent the stress tensor and strain tensor, respectively; $\alpha (\left|x-x^{\prime }\right|,\tau )$ is the nonlocal modulus, representing the nonlocal effect generated at position $x^{\prime }$ on another position x; $\tau =e_{0}a/l^{\prime }$, where $l^{\prime }$ is the external characteristic length, a is the internal characteristic length, and $e_{0}$ is an atomic simulation constant; *C_ijkl_* is the classical elasticity modulus tensor, and the subscripts *i*, *j* and *k*, *l* represent the directions of stress and strain, respectively.

The differential form of the nonlocal theory is popular in the analysis of nanostructures and for the one-dimensional nanobeam, it has the form of

(13) $$\sigma_{xx}-\mu \frac{\partial^{2}\sigma_{xx}}{dx^{2}}=E\varepsilon_{xx}$$

(14) $$\sigma_{xz}-\mu \frac{\partial^{2}\sigma_{xz}}{\partial x^{2}}=2G\varepsilon_{xz}$$

where $\sigma_{xx}$ and $\sigma_{xz}$ denote the normal stress and shear stress, respectively; $\mu ={\left(e_{0}a\right)}^{2}$ denotes the size scale parameter of the nanobeam.

Thus, the constitutive relationship of the nonlocal Levinson beam could be

(15) $$M^{L}-\mu \frac{\partial^{2}M^{L}}{\partial x^{2}}=EIk^{L}+EJ\lambda^{L}$$

(16) $$Q^{L}-\mu \frac{\partial^{2}Q^{L}}{\partial x^{2}}=GA\gamma^{L}+GI\eta^{L}$$

where $J=\frac{3}{20}Ih^{2}$.

By substituting Equations (15) and (16) into the transverse motion equations, Equations (8) and (9), the vibration equation of the nonlocal Levinson beam can be reached with

(17) $$\begin{array}{l} G\tilde{A^{L}}\left(\frac{\partial \varphi^{L}}{\partial x}+\frac{\partial^{2}w^{L}}{\partial x^{2}}\right)-P\frac{\partial^{2}w^{L}}{\partial x^{2}}+qw^{L}+\mu P\frac{\partial^{4}w^{L}}{\partial x^{4}}-\mu q\frac{\partial^{2}w^{L}}{\partial x^{2}}\\ =m_{0}\frac{\partial^{2}w^{L}}{\partial t^{2}}-\mu m_{0}\frac{\partial^{4}w^{L}}{\partial x^{2}\partial t^{2}} \end{array}$$

(18) $$\begin{array}{l} EI\frac{\partial^{2}\varphi^{L}}{\partial x^{2}}-c_{1}JE\left(\frac{\partial^{2}\varphi^{L}}{\partial x^{2}}+\frac{\partial^{3}w^{L}}{\partial x^{3}}\right)-G\tilde{A^{L}}\left(\varphi^{L}+\frac{\partial w^{L}}{\partial x}\right)\\ =m_{2}{}^{L}\frac{\partial^{2}\varphi^{L}}{\partial t^{2}}-\mu m_{2}{}^{L}\frac{\partial^{4}\varphi^{L}}{\partial x^{2}\partial t^{2}} \end{array}$$

where $\tilde{A^{L}}=\frac{2}{3}A$.

### 2.3. Governing Equation of Dynamic Stability of Nonlocal Levinson Beam

For a beam simply supported at both ends, its transverse and shear deformation can be defined as [32]

(19) $$w^{L}(x,t)=D(t)\sin\left(\frac{n\pi x}{l}\right)$$

(20) $$\varphi^{L}(x,t)=\chi (t)\cos\left(\frac{n\pi x}{l}\right)$$

where D and χ denote the time coordinates of $w^{L}$ and $\varphi^{L}$, respectively.

Substituting Equations (19) and (20) into the vibration governing equation of a nonlocal Levinson nanobeam (Equations (17) and (18)) gives

(21) $$\left[\begin{array}{l} -\frac{n\pi }{l}G\tilde{A^{L}}\chi (t)-{\left(\frac{n\pi }{l}\right)}^{2}G\tilde{A^{L}}D(t)+{\left(\frac{n\pi }{l}\right)}^{2}PD(t)+qD(t)+{\left(\frac{n\pi }{l}\right)}^{2}\mu qD(t)\\ +{\left(\frac{n\pi }{l}\right)}^{4}\mu PD(t)-m_{0}\frac{\partial^{2}D(t)}{\partial t^{2}}-\mu (\frac{n\pi }{l}{)}^{2}m_{0}\frac{\partial^{2}D(t)}{\partial t^{2}} \end{array}\right]\sin(\frac{n\pi x}{l})=0$$

(22) $$\left[\begin{array}{l} -{\left(\frac{n\pi }{l}\right)}^{2}EI\chi (t)+c_{1}{\left(\frac{n\pi }{l}\right)}^{2}EJ\chi (t)+c_{1}{\left(\frac{n\pi }{l}\right)}^{3}EJD(t)-G\tilde{A^{L}}\overset{}{\chi (t)-}\frac{n\pi }{l}G\overset{}{\tilde{A^{L}}D(t)}\\ -m_{2}{}^{L}\frac{\partial^{2}\chi (t)}{\partial t^{2}}-\mu (\frac{n\pi }{l}{)}^{2}m_{2}{}^{L}\frac{\partial^{2}\chi (t)}{\partial t^{2}} \end{array}\right]\cos(\frac{n\pi x}{l})=0$$

To satisfy Equations (21) and (22) at any time *t*, i.e., the items contained in the square bracket diminish, we have

(23) $$\left[\begin{array}{l} -\frac{n\pi }{l}G\tilde{A^{L}}\chi (t)-{\left(\frac{n\pi }{l}\right)}^{2}G\tilde{A^{L}}D(t)+{\left(\frac{n\pi }{l}\right)}^{2}PD(t)+qD(t)+{\left(\frac{n\pi }{l}\right)}^{2}\mu qD(t)\\ +{\left(\frac{n\pi }{l}\right)}^{4}\mu PD(t)-m_{0}\frac{\partial^{2}D(t)}{\partial t^{2}}-\mu {(}^{\frac{n\pi }{l})2}m_{0}\frac{\partial^{2}D(t)}{\partial t^{2}} \end{array}\right]=0$$

(24) $$\left[\begin{array}{l} -{\left(\frac{n\pi }{l}\right)}^{2}EI\chi (t)+c_{1}{\left(\frac{n\pi }{l}\right)}^{2}EJ\chi (t)+c_{1}{\left(\frac{n\pi }{l}\right)}^{3}EJD(t)-G\tilde{A^{L}}\overset{}{\chi (t)-}\frac{n\pi }{l}G\overset{}{\tilde{A^{L}}D(t)}\\ -m_{2}{}^{L}\frac{\partial^{2}\chi (t)}{\partial t^{2}}-\mu {\left(\frac{n\pi }{l}\right)}^{2}m_{2}{}^{L}\frac{\partial^{2}\chi (t)}{\partial t^{2}} \end{array}\right]=0$$

Equations (23) and (24) can be expressed in the matrix form:

(25) $$\begin{array}{cc} \; & \left[\begin{array}{cc} -m_{0}-\mu {\left(\frac{n\pi }{l}\right)}^{2}m_{0} & 0\\ 0 & -{m_{2}}^{L}-\mu {\left(\frac{n\pi }{l}\right)}^{2}{m_{2}}^{L} \end{array}\right]\cdot \left[\begin{array}{c} \ddot{D}\left(t\right)\\ \ddot{\chi }\left(t\right) \end{array}\right]+\\ \; & \left[\begin{array}{cc} -{\left(\frac{n\pi }{l}\right)}^{2}G\tilde{A^{L}}\;+q+{\left(\frac{n\pi }{l}\right)}^{2}\mu q & -\frac{n\pi }{l}G\tilde{A}\;\\ c_{1}{\left(\frac{n\pi }{l}\right)}^{3}EJ-\frac{n\pi }{l}G\tilde{A^{L}}\; & -{\left(\frac{n\pi }{l}\right)}^{2}EI+c_{1}{\left(\frac{n\pi }{l}\right)}^{2}EJ-G\tilde{A^{L}}\; \end{array}\right]\cdot \left[\begin{array}{c} D(t)\\ \chi (t) \end{array}\right]\\ \; & -\left[\begin{array}{cc} -{\left(\frac{n\pi }{l}\right)}^{2}-{\left(\frac{n\pi }{l}\right)}^{4}\mu & 0\\ 0 & 0 \end{array}\right]P\left[\begin{array}{c} D(t)\\ \chi (t) \end{array}\right]=0 \end{array}$$

i.e.,

(26) $$M^{L}\ddot{d}+\{K_{e}{}^{L}-P(t)K_{g}{}^{L}\}d=0$$

where

$$M^{L}=\left[\begin{array}{cc} a_{1}{}^{L} & a_{2}{}^{L}\\ a_{3}{}^{L} & a_{4}{}^{L} \end{array}\right],\;K_{e}{}^{L}=\left[\begin{array}{cc} a_{5}{}^{L} & a_{6}{}^{L}\\ a_{7}{}^{L} & a_{8}{}^{L} \end{array}\right],\;K_{g}{}^{L}=\left[\begin{array}{cc} a_{9}{}^{L} & 0\\ 0 & 0 \end{array}\right],\;d=\left\{\begin{array}{c} D(t)\\ \chi (t) \end{array}\right\}$$

are the matrices of mass, stiffness, geometric stiffness and the deformation vector, with $a_{1}{}^{L}=-m_{0}-\mu {\left(\frac{n\pi }{l}\right)}^{2}m_{0}$, $a_{2}{}^{L}=0$, $a_{3}{}^{L}=0$, $a_{4}{}^{L}=-m_{2}{}^{L}-\mu {\left(\frac{n\pi }{l}\right)}^{2}m_{2}{}^{L}$, $a_{5}{}^{L}=-{\left(\frac{n\pi }{l}\right)}^{2}G\tilde{A^{L}}+q+{\left(\frac{n\pi }{l}\right)}^{2}\mu q$, $a_{6}{}^{L}=-\frac{n\pi }{l}G\tilde{A^{L}}$, $a_{7}{}^{L}=c_{1}{\left(\frac{n\pi }{l}\right)}^{3}EJ-\frac{n\pi }{l}G\tilde{A^{L}}$, $a_{8}{}^{L}=-{\left(\frac{n\pi }{l}\right)}^{2}EI+c_{1}{\left(\frac{n\pi }{l}\right)}^{2}EJ-G\tilde{A^{L}}$, $a_{9}{}^{L}=-{\left(\frac{n\pi }{l}\right)}^{2}-{\left(\frac{n\pi }{l}\right)}^{4}\mu$.

The axial excitation P(t) can be expressed as

(27) $$P(t)=[\alpha +\beta \cos(\theta t)]P_{cr}{}^{L}$$

where α and β denote the ratios of the static and dynamic components of P(t) to the Euler buckling load *P_cr_*, respectively; θ denotes the excitation frequency.

Thus, Equation (26) can be rewritten as

(28) $$M^{L}\ddot{d}+\{K_{e}{}^{L}-[[\alpha +\beta \cos(\theta t)]P_{cr}{}^{L}]K_{g}{}^{L}\}d=0$$

Equation (28) is a type of Mathieu–Hill equation with periodic coefficients. The distribution of the solutions of the Mathieu–Hill equation on a parametric plane is divided by regions of stable solutions and unstable solutions, and the boundaries of these two types of regions can be fixed by solving the eigenvalue problem corresponding to Equation (28). Among the regions of dynamic stability, the first region corresponding to the first-order eigenvalue has the largest width and represents the most dangerous region, called the principal region of dynamic instability. The boundary of the principal instability region (PIR) could be obtained by solving the eigenvalue problem [33].

(29) $$\left|K_{e}{}^{L}-P_{cr}{}^{L}(\alpha \pm \frac{\beta }{2})\cdot K_{g}{}^{L}-\frac{\theta^{2}}{4}M^{L}\right|=0$$

**Figure 2:**
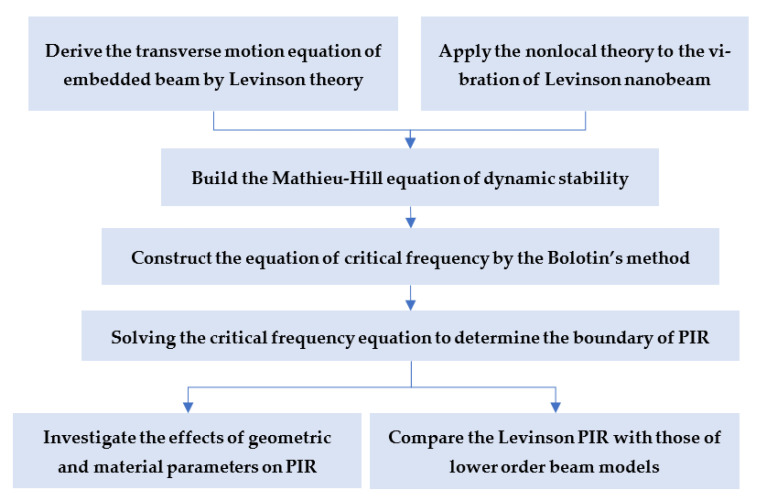
Flow chart of dynamic stability analysis on the Levinson nanobeam.

Consequently, the study of the dynamic stability of the Levinson nanobeam can be carried out corresponding to the flow chart given in Figure 2.

## 3. Results and Discussions

**Figure 3:**
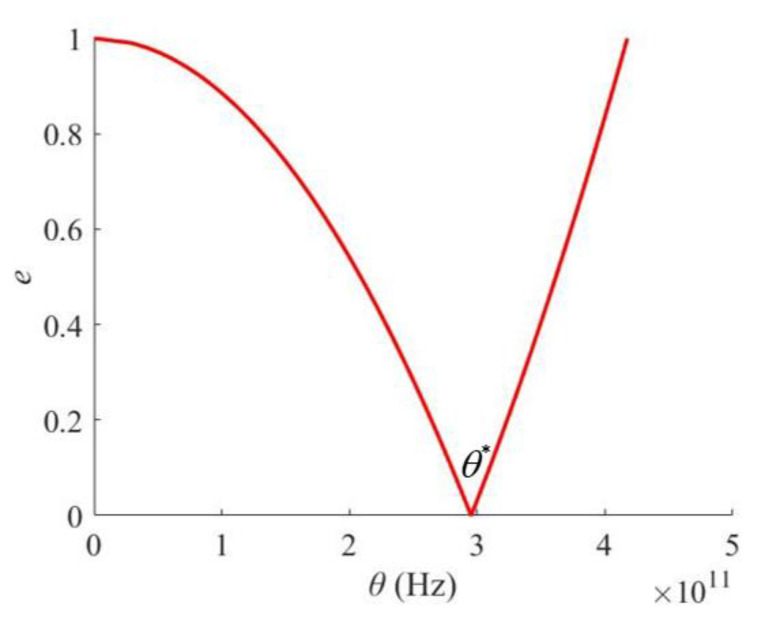
The principal instability region (PIR) of the Levinson nanobeam.

A Levinson-type nanobeam with the parameters shown in Table 1 is investigated in this section, and the parameter values are set according to the literature [34]. According to the geometric and material properties indicated in Table 1, the boundaries of the principle instability region (PIR) can be obtained by solving Equation (29) and are demonstrated on a parametric plane (*e*, theta) as shown in Figure 3 (the red line), where *e* = *β*/2(1−*α*) denotes the amplitude parameter of excitation. The computation of the PIR and the drawing of all figures in this section were carried out on the platform of MATLAB. In Figure 3, the area enclosed by the left and right boundaries represents the principal region of dynamic instability (PIR). That is, if the point corresponding to the excitation parameters *α*, *β,* and *θ* is located inside the PIR, the nanobeam would be in the state of dynamic instability, or if the point is located outside the PIR, the dynamic state of the nanobeam could be stable.

Meanwhile, the intersection of the boundaries of the PIR on the horizontal ordinate axis, $\theta^{*}$, is the central critical frequency of excitation for dynamic instability, so $\theta^{*}$ represents the position of the PIR on the parametric plane. Therefore, the determination of the boundaries of the PIR are crucial for evaluating the dynamic stability of the Levinson nanobeams.

**Figure 4:**
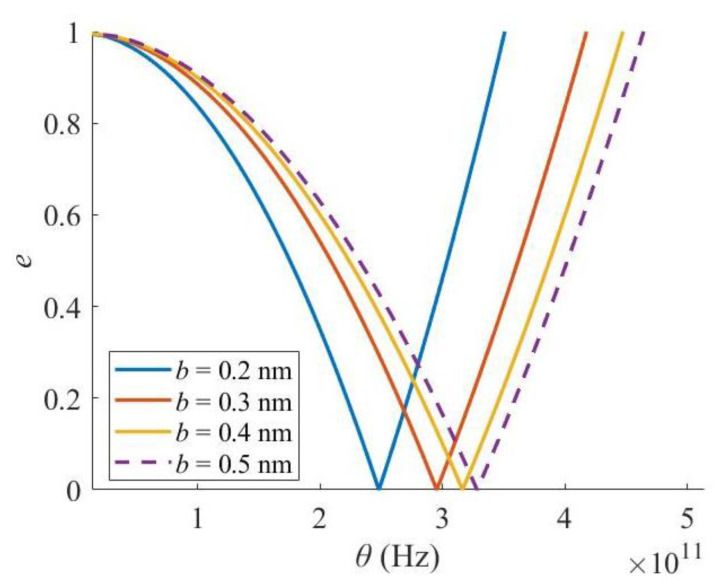
Effect of cross-sectional width *b*.

The influence of the cross-sectional width *b* of the Levinson nanobeam on the width of the principal instability region (PIR) is shown in Figure 4. It is indicated that for the Levinson nanobeam embedded in a medium, the increment in width makes the critical frequency $\theta^{*}$ move to the higher frequency zone, and the width of the PIR enlarges. However, the effect gets weaker when its value becomes larger. It is noteworthy that the abscissa here is only the excitation frequency, not the ratio of excitation frequency to 2 times the natural frequency of a beam commonly set in the literature. The reason for such operation is to demonstrate the effects of various parameters on the excitation frequency, excluding the natural frequency.

**Figure 5:**
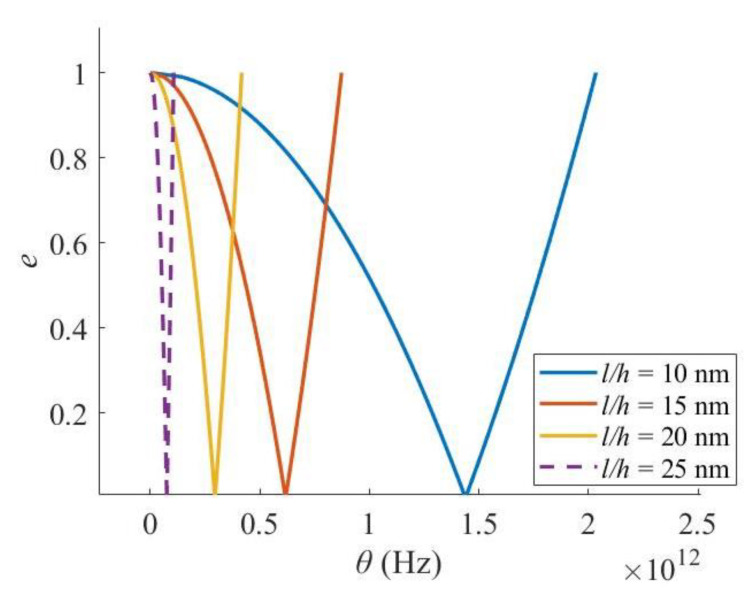
Effect of length/height ratio.

Comparatively, when the length/height ratio increases (the length increases or the height decreases), i.e., the nanobeam becomes slenderer, the critical frequency of excitation moves to the lower frequency zone, and the width of the PIR shrinks (Figure 5). Hence, when the nanobeam becomes slenderer, the possibility of dynamic instability decreases, while it would be unstable under a smaller excitation frequency. Moreover, the length/height ratio has a much more significant effect than the sectional width.

**Figure 6:**
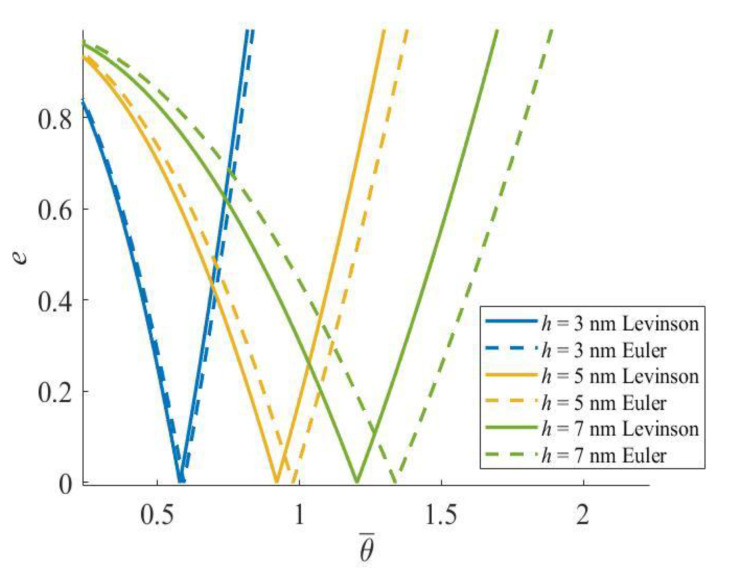
Comparison of PIR between the Levinson and Euler nanobeams.

In order to demonstrate the necessity of a higher-order beam model under larger sectional heights, the PIR boundaries from the Levinson and Euler–Bernoulli beam theories are compared in Figure 6, where the excitation frequency is nondimensionalized by $\bar{\theta }=\theta L\sqrt{\rho /E}$. It is clearly shown that, when the height is relatively small, the boundaries of both beam theories are close, but when the height increases, the widths and positions of the PIRs from the two beam theories become different.

**Figure 7:**
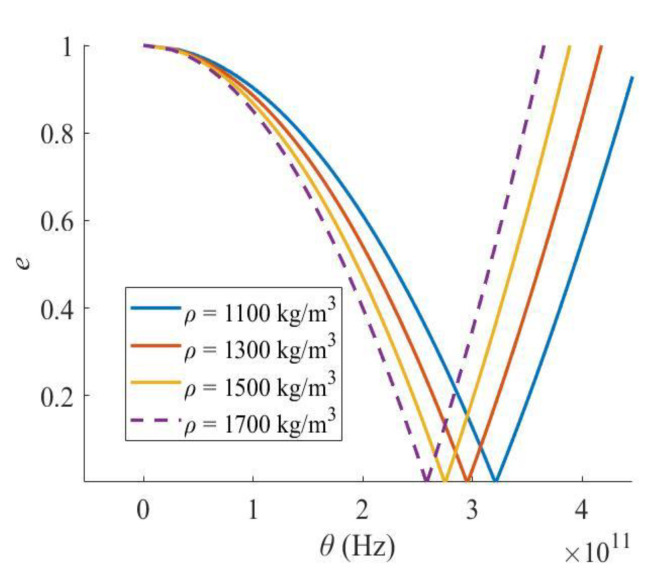
Effect of mass density.

If the mass density of the Levinson nanobeam increases, the critical excitation frequency and the width of the PIR also reduces (Figure 7), so the increment in density would enhance the dynamic stability of the Levinson nanobeam if better performance in dynamic stability is judged by a smaller excitation frequency and a narrower PIR width.

**Figure 8:**
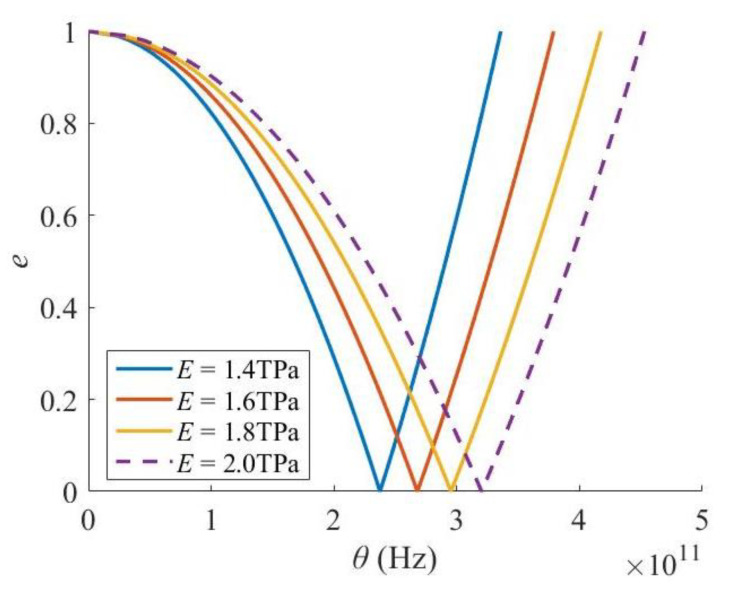
Effect of Young’s modulus.

**Figure 9:**
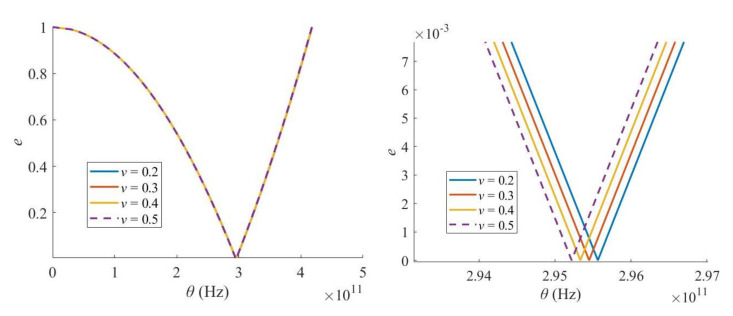
Effect of Poisson’s ratio.

In contrast, when the Young’s modulus of a Levinson nanobeam increases, both the critical excitation frequency and the width of the PIR increase (Figure 8), i.e., the dynamic stability of the Levinson nanobeam gets worse. In addition, the Poisson’s ratio has little effect on the PIR (Figure 9).

**Figure 10:**
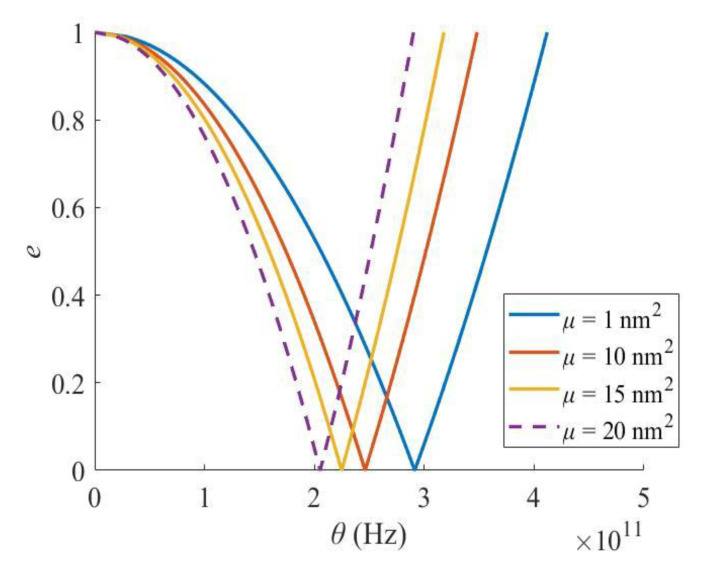
Effect of size scale parameter.

Furthermore, with the increase in the size scale parameter, both the critical excitation frequency and the width of PIR decreases (Figure 10). Therefore, neglecting the small-scale effect of a nanobeam would overestimate its dynamic stability performance, and the nonlocal model should be used for evaluating the dynamic stability of a nanobeam.

**Figure 11:**
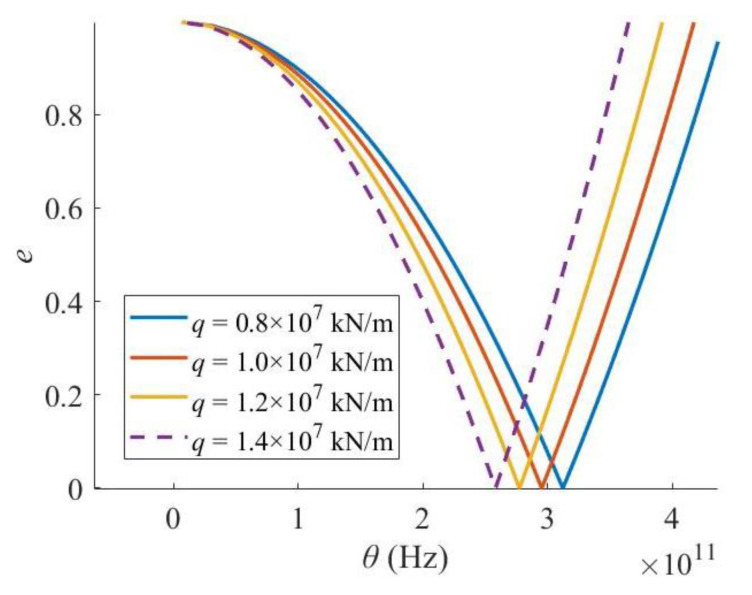
Effect of medium stiffness.

When the Levinson nanobeam is embedded in a stiffer medium, the critical excitation frequency and the width of the PIR also reduces (Figure 11), i.e., the elastic foundation enhances the dynamic stability performance of the Levinson nanobeam.

In summary, when any of the above parameters change, the value of critical excitation frequency and the width of the PIR vary in the same direction, i.e., both the critical excitation frequency and the width of the PIR become smaller or larger. When the width, length/height ratio, density, Young’s modulus, Poisson’s ratio, size scale parameter, and medium stiffness increase by about 1.5 times, the width of the PIR varies by about 19%, −57%, −20%, 65%, 0, −9%, and −11%, respectively. To reduce the critical excitation frequency and the width of the PIR, the Levinson nanobeam should have a smaller cross-sectional width and height or Young’s modulus and a larger length, density, and foundation stiffness. According to the theory of dynamic stability, the critical excitation frequency is basically two times the natural frequency of the loaded beam. Since smaller cross-sectional dimensions or Young’s moduli lead to higher natural frequencies, and larger lengths, densities, and foundation stiffnesses could result in smaller natural frequencies, the critical excitation frequency would become smaller when these parameters vary in the above directions. Moreover, the higher-order Levinson beam theory achieves a more accurate boundary of the PIR than the lower-order Euler–Bernoulli beam theory when the sectional height of the nanobeam is larger.

## 4. Conclusions

The dynamic instability of a nanobeam subjected to an elastic foundation and axial harmonic excitation is investigated using the Levinson beam model. The transverse vibration equations of the embedded Levinson nanobeam are derived and the nonlocal form of the vibration equations are built using Eringen’s theory. The boundaries of the principal instability region (PIR) of the Levinson nanobeam are determined using the Bolotin method.

The value of critical excitation frequency and the width of the PIR would be reduced under smaller sectional dimensions and Young’s moduli and larger lengths, densities, and foundation stiffnesses. This is owing to the fact that the critical excitation frequency of dynamic instability is commonly two times the natural frequency, while the increments in cross-sectional dimensions and Young’s moduli enlarge the natural frequency, and the increments in the length, density and foundation stiffness decrease the natural frequency. For the specific nanobeam studied, when the width, length/height ratio, density, Young’s modulus, Poisson’s ratio, size scale parameter, and medium stiffness increase by about 1.5 times, the width of the PIR changes by about 19%, −57%, −20%, 65%, 0, −9%, and −11%, respectively.

The length/height ratio of the nanobeams has a more obvious effect on the PIR, and the Poisson’s ratio has little effect. When the sectional height is larger, the Levinson beam model produces better results of the PIR than the Euler–Bernoulli beam theory. Additionally, ignoring the small-scale effect of the Levinson nanobeam would overestimate the dynamic stability performance.

The proposed method can be used for the dynamic stability evaluation of nanobeams with smaller length/height ratios to produce higher accuracy than the Timoshenko beam model, which have been typically employed for such nanobeams in previous studies. The shear correction coefficient adopted by the Timoshenko beam model would fluctuate with the changes in the structural aspect ratio and result in calculation errors, while this is no longer required in the Levinson beam model, since the shear strain disappears at the top and bottom of the beam.

## Figures and Tables

**Table 1:** Geometric and material parameters of the Levinson nanobeam.

Material Parameters		Geometric Parameter	
*E* (TPa)	1.8	*l* (nm)	20
*ρ* (kg/m^3^)	1300	*h* (nm)	1
υ	0.5	*b* (nm)	0.3
*q* (GPa)	0.1	*μ* (nm^2^)	0.3

## Data Availability

The research data can be found in the paper.
